# Supplementary material for: Dephosphocholination by Legionella effector Lem3 functions through remodelling of the switch II region of Rab1b
Source: Nat Commun. 2023 Apr 19;14:2245. doi: 10.1038/s41467-023-37621-7 (PMC10115812; doi:10.1038/s41467-023-37621-7)
Supplement: Supplementary file 1 — Supplementary Information [file 41467_2023_37621_MOESM1_ESM.pdf]

Supplementary Information

Dephosphocholination by Legionella effector Lem3 functions through remodelling of the switch II region of Rab1b

Table of contents

Supplementary Figures

Supplementary Fig. 1: Structural relationship of Lem3 to PPM1A and SidD

Supplementary Fig. 2: Modification of Rab1b with different PTMs

Supplementary Fig. 3: Sequential and structural comparison of Rab1b and Rab35

Supplementary Fig. 4: Optimisation of complex formation

Supplementary Fig. 5: Substrate and metal ion coordination of the Lem3:Rab1b complex$_{T391C}$

Supplementary Fig. 6: Molecular interactions of the Lem3:Rab1b complex$_{T391C}$

Supplementary Tables

Supplementary Table 1: X-ray data collection and refinement statistics

Supplementary Figures

Supplementary Figure 1

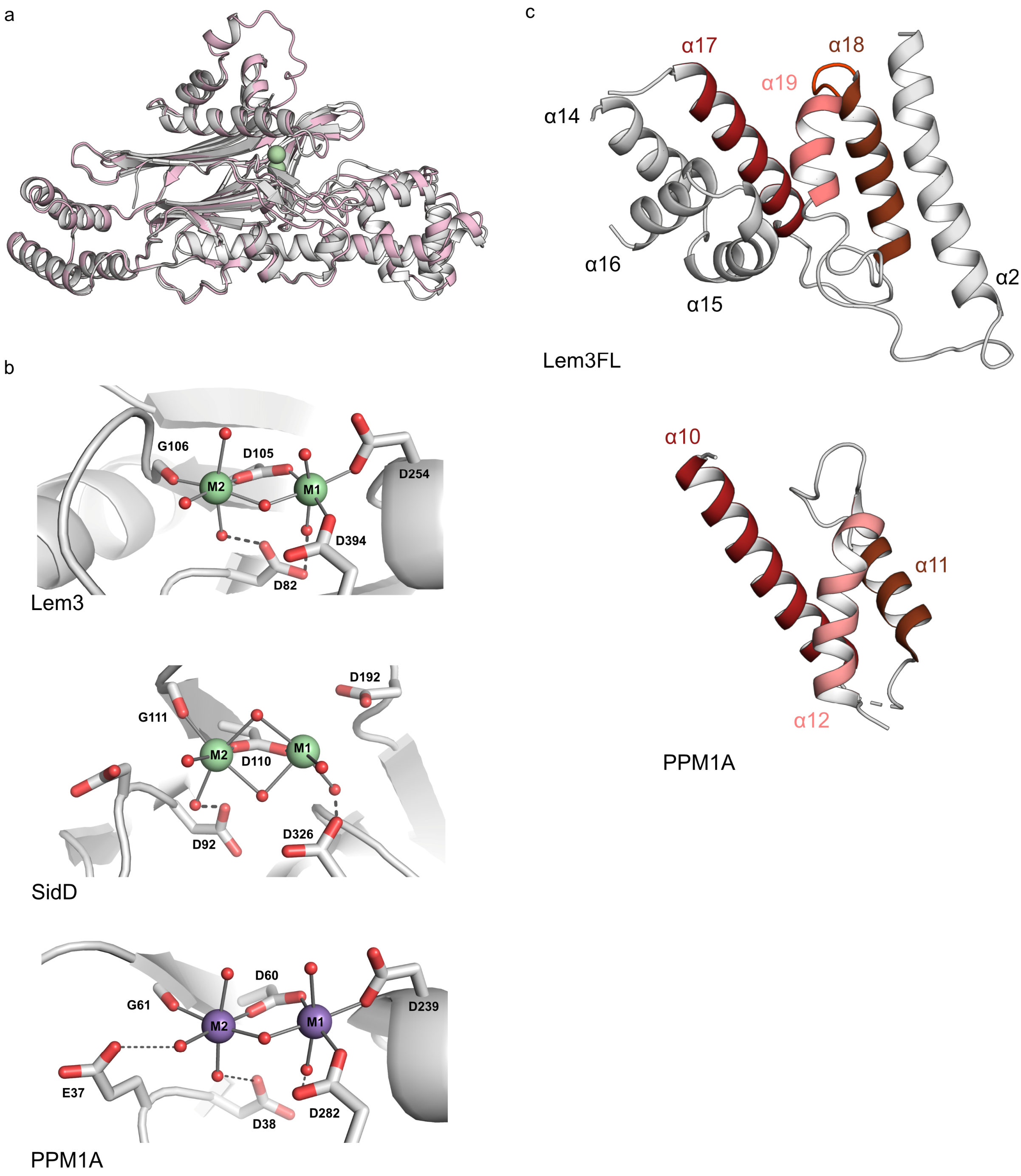

Supplementary Fig. 1: Structural relationship of Lem3 to PPM1A and SidD
(**a**) Superimposition of Lem3$_{21\text{-}486}$ (PDB ID: 8ANP) crystal structure (Grey) with metal ions (spheres, Green) and Lem3$_{21\text{-}486}$ Alphafold2 model (Pink). (**b**) Comparative depiction of positions of aspartate residues of Lem3 (PDB ID: 8ANP), PP1MA (PDB ID: 4RA2) and SidD (PDB ID: 6RRE) contributing to metal ion coordination. The arrangement of respective aspartate residues and metal ions is depicted as sticks and spheres (Mg^{2+}, Green; Mn^{2+}, Purple). (**c**) Cartoon representation of C-terminal bundles of Lem3$_{FL}$ (PDB ID: 8AGG) and PPM1A (PDB ID: 4RA2).

Supplementary Figure 2

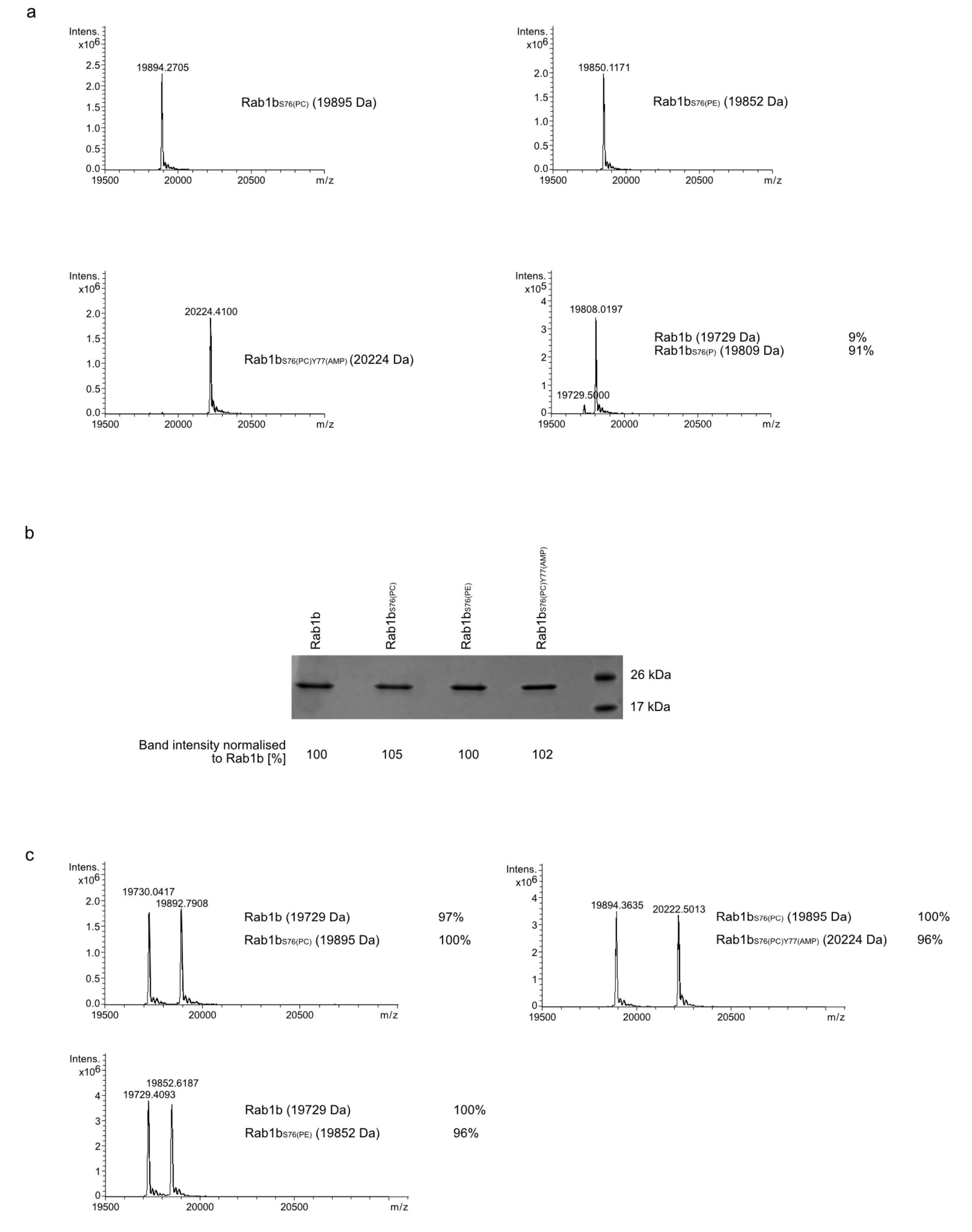

Supplementary Fig. 2: Modification of Rab1b with different PTMs

(**a**) MS-based quality control of post-translationally modified Rab1b proteins. (**b**) SDS-PAGE of purified Rab1b, Rab1b$_{S76(PC)}$, Rab1b$_{S76(PE)}$ and Rab1b$_{S76(PC)Y77(AMP)}$ to assure comparable amounts for comparative MS analysis. 500 µg of each protein were loaded and amounts were quantified according to band intensity. (**c**) MS analysis of equimolar protein mixtures. Peak intensities were compared relative to each other.

Supplementary Figure 3

a

```
Rab1b   1 M N P E Y D Y L F K L L L I G D S G V G K S C L L L R F A D D T Y T E S Y I S T I G V D F K I R T I E L D G K T I K L Q 60
Rab35   1 M A R D Y D H L F K L L I I G D S G V G K S S L L L R F A D N T F S G S Y I T T I G V D F K I R T V E I N G E K V K L Q 60
          *     : * * : * * * * * : * * * * * * * * * . * * * * * * * : * : :   * * * : * * * * * * * * * * : * : : * : . : * * *

Rab1b  61 I W D T A G Q E R F R T I T S S Y Y R G A H G I I V V Y D V T D Q E S Y A N V K Q W L Q E I D R Y A S E N V N K L L V G 120
Rab35  61 I W D T A G Q E R F R T I T S T Y Y R G T H G V I V V Y D V T S A E S F V N V K R W L H E I N Q N C - D D V C R I L V G 119
          * * * * * * * * * * * * * * * : * * * * : * * : * * * * * * * .   * * : . * * * : * * : * * : :   .   : : *   : : * * *

Rab1b 121 N K S D L T T K K V V D N T T A K E F A D S L G I P F L E T S A K N A T N V E Q A F M T M A A E I K K R M G P G A A S G 180
Rab35 120 N K N D D P E R K V V E T E D A Y K F A G Q M G I Q L F E T S A K E N V N V E E M F N C I T E L V L R A K K D N L A K Q 179
          * * . *       : * * * : .     *   : * * . . : * *   : : * * * * * :   . * * * :   *     : :     :   :         .   * .

Rab1b 181 - G E R P N L K I D S T P V K P A G G G C C
Rab35 180 Q Q Q Q Q N D V V K L T K N S K R K K R C C
              : :   *     : .   *     .           * *
```

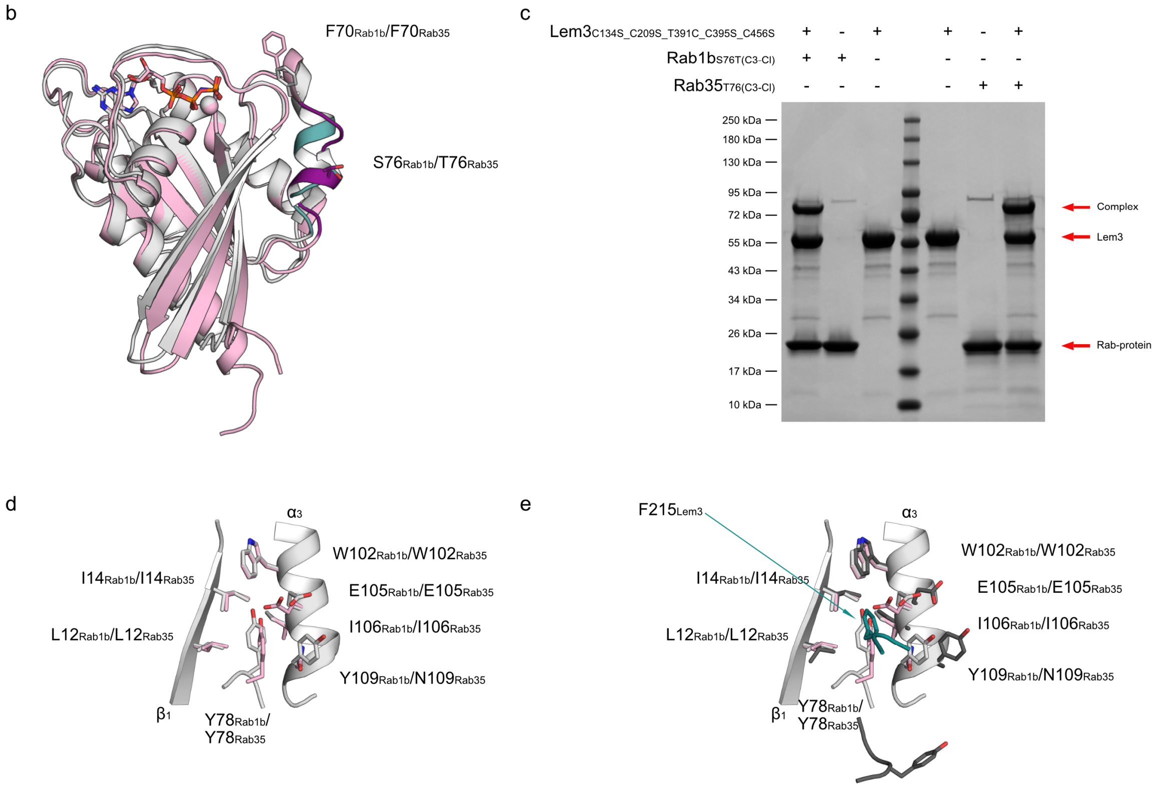

Supplementary Fig. 3: Sequential and structural comparison of Rab1b and Rab35

(**a**) Sequence alignment of Rab1b and Rab35. Asterisks indicate fully conserved residues, colons indicate conservation between groups of strongly similar properties and periods indicate conservation between groups of weakly similar properties. $F70_{Rab1b/Rab35}$ (Blue) and the octapeptide sequence recognized by AnkX (Pink) are highlighted. The respective phosphocholinated amino acid is underlined. (**b**) Superimposition of Rab1b (PDB ID: 3NKV) (Grey) and Rab35 (PDB ID: 6IF3) (Pink), $F70_{Rab1b/Rab35}$, $S76_{Rab1b}$ and $T76_{Rab35}$ are represented as sticks. The respective switch II region is highlighted in cyan (Rab1b) and magenta (Rab35). (**c**) Complex formation between $Lem3_{C134S\_C209S\_T391C\_C395S\_C456S}$ and $Rab1b_{S76T(PC\text{-}Cl)}$ or $Rab35_{T76(PC\text{-}Cl)}$ analysed by SDS-PAGE Gel shift assay. (**d-e**) Cartoon representation of Rab1b (PDB ID: 3NKV) (Grey), residues of the hydrophobic core are shown as sticks. Corresponding Rab35 (PDB ID: 6IF3) residues are superimposed and shown as sticks (Pink) (d). Cartoon representation of Lem3 thorn with $F215_{Lem3}$ at the tip (Green). Lem3-bound Rab1b amino acids interacting with $F215_{Lem3}$ are shown as sticks (Darkgrey) (e).

Supplementary Figure 4

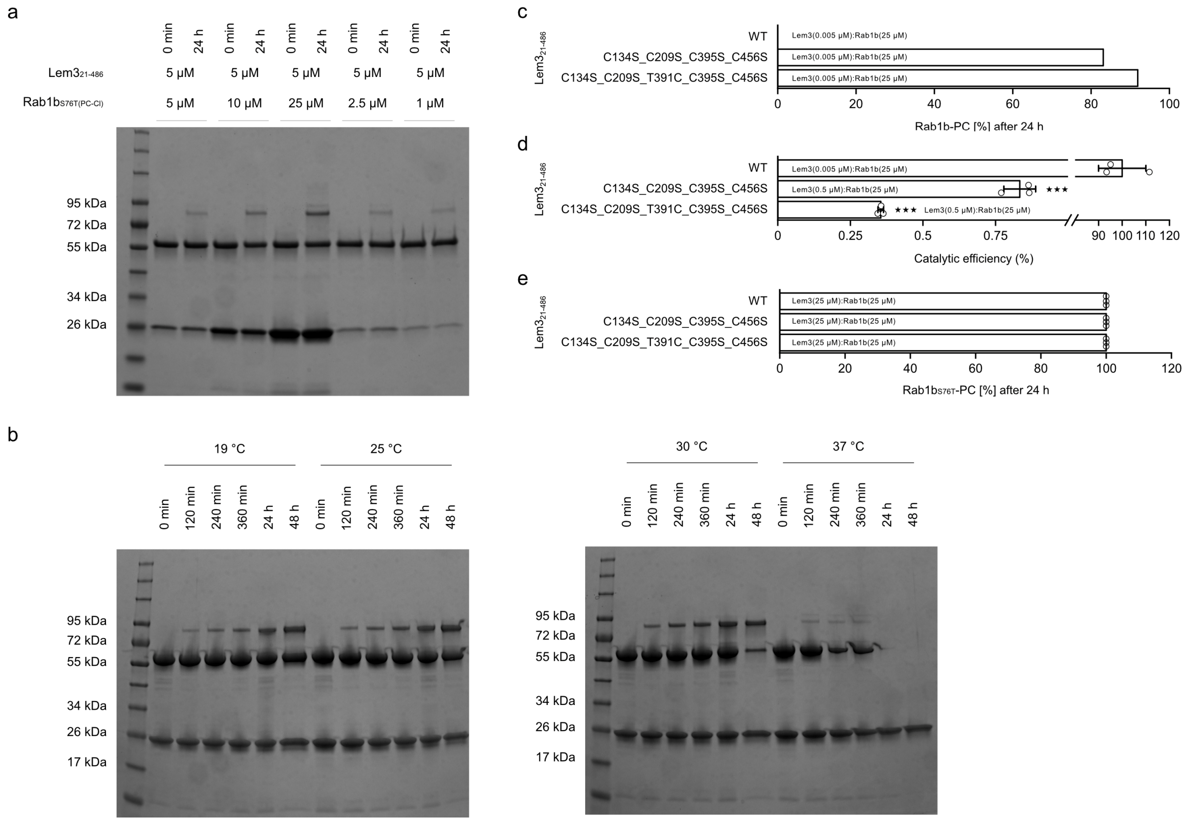

Supplementary Fig. 4: Optimisation of complex formation

(**a**) SDS-PAGE gel shift assay to analyse protein concentration ratio dependence of complex formation. Stated amounts of Lem3 and Rab1b$_{S76T(PC\text{-}Cl)}$ were incubated at 19°C, samples were taken at indicated time points. (**b**) SDS-PAGE gel shift assay to assess complex formation at different temperatures and over time. Equimolar amount of Lem3$_{21\text{-}486}$ and Rab1b$_{S76T(PC\text{-}Cl)}$ were incubated at stated temperatures and samples were taken at indicated time points. (**c**) Activity assay for Lem3 cysteine to serine mutants. Percentage of Rab1b$_{S76(PC)}$ demodification was monitored after 24 h. (**d**) Catalytic efficiency of Lem3 cysteine to serine mutants on Rab1b$_{S76(PC)}$. Catalytic efficiencies were determined from MS-derived dephosphocholination curves. Means (±SD) represent three independent biological replicates (unpaired, two-tailed t-test; ***: all p values equal less than 0.0001). (**e**) Activity assay for Lem3 cysteine to serine mutants. Percentage of Rab1b$_{S76T(PC)}$ was monitored after 24 h.

Supplementary Figure 5

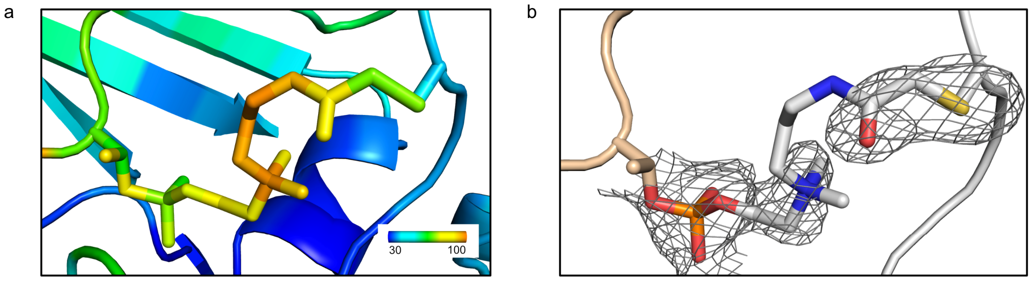

c

Phosphatase	aa	Organism	PDB ID
Lem3	L68	*L. pneumophila*	8ANP
SidD	F74	*L. pneumophila*	6RRE
PPM1A	R33	*H. sapiens*	6B67
PPM1B	R33	*H. sapiens*	2P8E
PPM1H	K87	*H. sapiens*	7KPR
PPM1K	R104	*H. sapiens*	4DA1
SpoIIE	K602	*B. subtilis*	5MQH
RsbX	K19	*B. subtilis*	3W41
DSM 20745	K17	*S. thermophilus*	3PU9
tPhpA	R13	*T. elongates*	2Y09
MtPstP	R20	*M. tuberculosis*	2CM1
MspP	R17	*M. smegmatis*	2V06
PppA	R21	*P. aeruginosa*	6JKV
PPM2C	R441	*B. taurus*	6V0T
MspP	R17	*M. smegmatis*	2JFR
SaSTP	R13	*S. agalactiae*	2PK0
Stp1	R14	*S. aureus*	5F1M
Phosphatase	R40	*T. gondii*	2I44
FEM-2	R172	*C. elegans*	4JND
OsPP2C06	R159	*O. sativa*	4OIC
OsPP2C50	S70	*O. sativa*	5ZCG
HAB1	R199	*A. thaliana*	4DS8
PP2CA	R114	*A. thaliana*	4N0G
TAP38/PPH1	R69	*A. thaliana*	4YZG
ABI1	R138	*A. thaliana*	3NMN
ABI2	R122	*S. cerevisiae*	3UJK
TAP1	H47	*M. musculus*	5NZZ

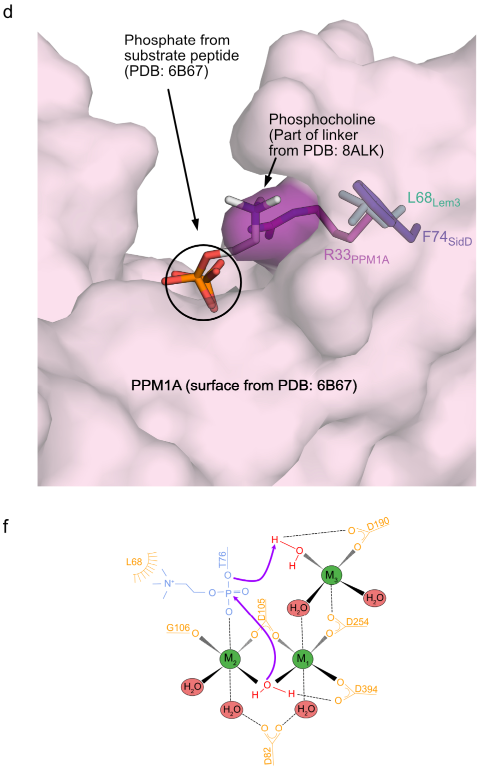

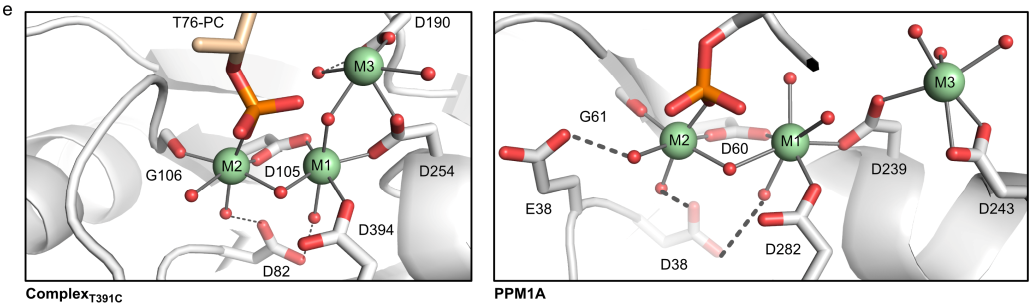

Supplementary Fig. 5: Substrate and metal ion coordination of the Lem3:Rab1b complex$_{T391C}$
(**a**) B-factors of linker atoms are considerably higher in comparison to the surrounding amino acids (scale bar unit: Å^2) . (**b**) Linker density from the unbiased omit electron density map contoured at 2.5 σ**.** (**c**) Structural alignment of members of the PPM family in humans and PPM phosphatase shaped proteins with available structures found by structure comparison via Pfam (Accession numbers: PF00481 and PD13672, doi: 10.1093/nar/gkaa913). The list contains residues that are conserved in function (but not necessarily in amino acid sequence) to R33 in the canonical PPM phosphatase PPM1A. (**d**) Surface representation of PPM1A (PDB ID: 6B67), R33$_{PPM1A}$ and bound phosphate shown as sticks. Lem3 (PDB ID: 8ALK) and SidD (PDB ID: 6RRE) are superimposed to PPM1A (PDB ID. 4RA2) with only L68$_{Lem3}$ and F74$_{SidD}$ shown. (**e**) Metal ion coordination of complex$_{T391C}$ (the choline and the rest of the linker is not represented for clarity) and PPM1A (PDB ID: 6B67). (**f**) Schematic depiction of the putative dephosphocholination mechanism catalysed by Lem3. Purple represent the nucleophilic attacks in the reaction.

Supplementary Figure 6

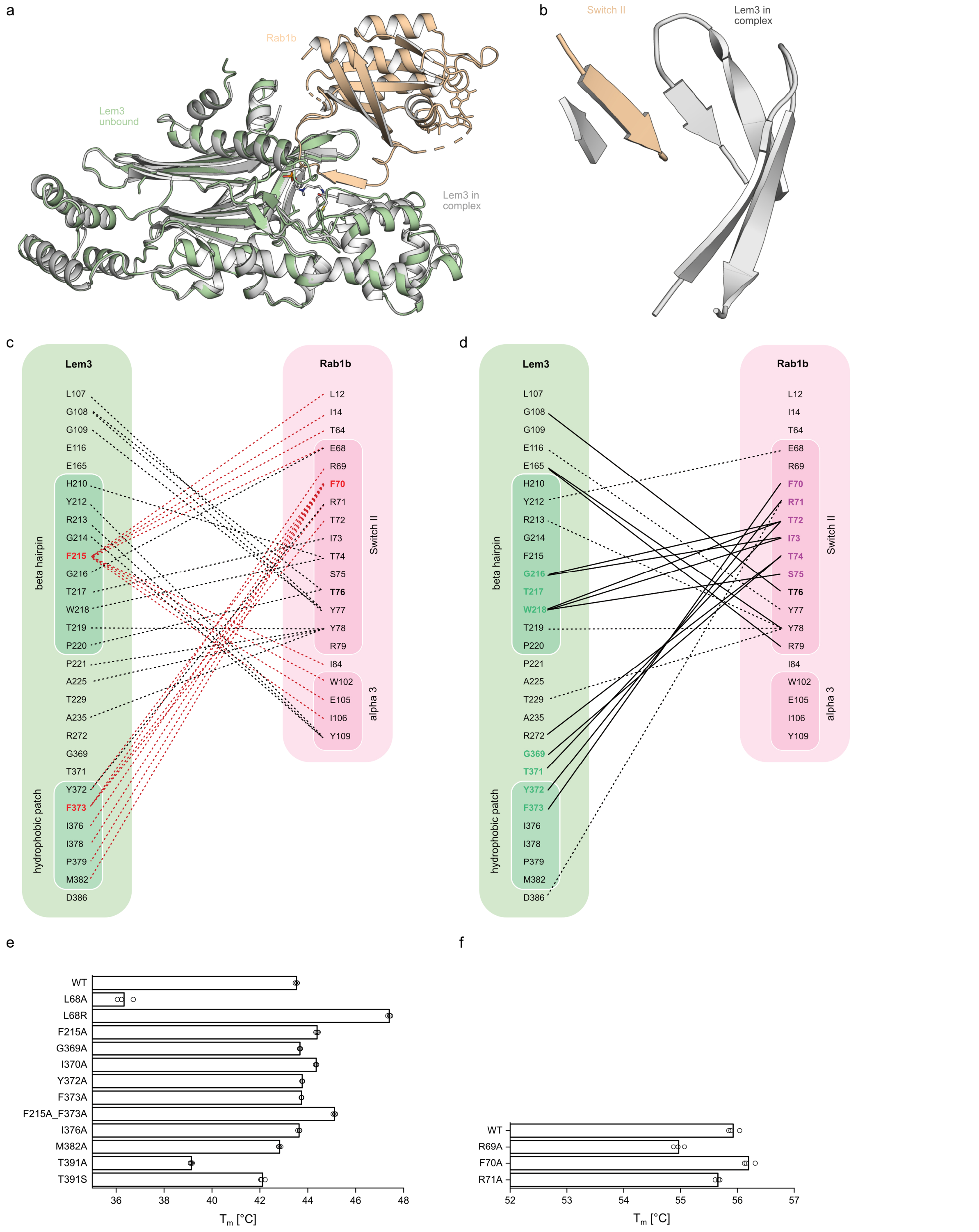

Supplementary Fig. 6: Molecular interactions of the Lem3:Rab1b complex$_{T391C}$

(**a**) Superimposition of complex$_{T391C}$ (PDB ID: 8ALK) and Lem3$_{21-486}$ (PDB ID: 8ANP). **(b)** Cartoon representation of newly formed U-shaped intramolecular β-sheet (Lem3 β-strands (Grey), Rab1b switch II β-strand (Wheat)). **(c** and **d)** Overall interaction profile of Lem3 with Rab1b as determined from the complex$_{T391C}$ structure. Hydrophobic interactions are represented by dashed lines in **c** and coloured in red when involving the three crucial phenylalanine residues. Polar interactions are represented in **d**. (**e** and **f**) Melting points of all Lem3 (e) and Rab1b (f) constructs. The experiment was performed in three independent replicates (each one is represented by a circle). Data are presented as mean values.

Supplementary Table

Supplementary Table 1: X-ray data collection and refinement statistics

	$Lem3_{21-486}$	**$Lem3_{FL}$**	**Lem3:Rab1**
**Crystal parameters**			
Space group	$P2_12_12_1$	$P6_122$	$P6_1$
Cell constants	a=55.7 Å<br>b=187.7 Å<br>c=193.0 Å	a=b=78.02 Å<br>c=382.01 Å	a=b=119.33 Å<br>c=79.06 Å°
Subunits / AU[a]	4	1	1
**Data collection**			
Beamline	X06SA, SLS	P13, EMBL, DESY	P13, EMBL, DESY
Wavelength (Å)	0.979	0.97624	0.979124
Resolution range (Å)[b]	30-2.2 (2.3-2.2)	67.6 - 3.6 (3.94- 3.60)	103.3 - 2.15 (2.23- 2.15)
No. observations	787 494	213 420	476 566
No. unique reflections[c]	103 694	8 684	34 977
Multiplicity [b]	4.0 (4.1)	24.6 (24.6)	13.6 (12.8)
Completeness (%)[b]	99.7 (99.8)	98.7 (99.7)	100 (100)
R_{merge} (%)[b, d]	9.4 (67.2)	17.0 (107.9)	16.1 (115.7)
$CC_{1/2}$ [b]	0.997 (0.695)	0.999 (0.673)	0.995 (0.761)
I/σ (I)[b]	11.5 (2.1)	14.2 (3.8)	9.7 (2.3)
**Refinement**			
Resolution range (Å)	15-2.2	66.5-3.6	103.3-2.15
No. refl. working set	98 136	8 266	33 102
No. refl. test set	5 165	409	1 839
No. non-hydrogen atoms	13814	3843	4940
Ligand	0	0	16
Metal ions	7	1	4
Solvent	271	0	72
R_{work}/R_{free} (%)[e]	18.9/22.9	26.2/29.8	18.1/22.7
r.m.s.d. bond (Å) / (angle)[f]	0.002/1.2	0.001/0.38	0.003/0.52
Average B-factors ($Å^2$)	51.9	171.3	59.1
Macromolecules	56.0	171.3	59.0
Ligand	N.A.	N.A.	86.0
Metal ions	46.9	196.3	67.4
Solvent	49.1	N.A.	53.0
Ramachandran Plot (%)[g]	98.4/1.6/0	95.4/4.6/0	98.5/1.5/0
PDB accession code	**8ANP**	**8AGG**	**8ALK**

[a] Asymmetric unit

[b] The values in parentheses correspond to the highest resolution shell

[c] Data reduction was carried out with XDS and from a single crystal. Friedel pairs were treated as identical reflections

[d] $R_{merge}(I) = \Sigma_{hkl}\Sigma_j \mid I(hkl)_j - \langle I(hkl)\rangle \mid / \Sigma_{hkl} \Sigma_j I(hkl)_j$, where $I(hkl)_j$ is the jth measurement of the intensity of reflection hkl and $\langle I(hkl)\rangle$ is the average intensity

[e] $R = \Sigma_{hkl} \mid |F_{obs}| - |F_{calc}| \mid / \Sigma_{hkl} |Fobs|$, where R_{free} is calculated without a sigma cut-off for a randomly chosen 5% of reflections, which were not used for structure refinement, and R_{work} is calculated for the remaining reflections

[f] Deviations from ideal bond lengths/angles

[g] Percentage of residues in favoured region / allowed region / outlier region

N.A. (not applicable)
