# Supplementary material for: Dephosphocholination by Legionella effector Lem3 functions through remodelling of the switch II region of Rab1b
Source: Nat Commun. 2023 Apr 19;14:2245. doi: 10.1038/s41467-023-37621-7 (PMC10115812; doi:10.1038/s41467-023-37621-7)
Supplement: Supplementary file 3 — Reporting Summary [file 41467_2023_37621_MOESM3_ESM.pdf]

nature portfolio

Corresponding author(s): Aymelt Itzen

Last updated by author(s): Mar 13, 2023

Reporting Summary

Nature Portfolio wishes to improve the reproducibility of the work that we publish. This form provides structure for consistency and transparency in reporting. For further information on Nature Portfolio policies, see our Editorial Policies and the Editorial Policy Checklist.

Statistics

For all statistical analyses, confirm that the following items are present in the figure legend, table legend, main text, or Methods section.

n/a	Confirmed	
☐	☒	The exact sample size (*n*) for each experimental group/condition, given as a discrete number and unit of measurement
☐	☒	A statement on whether measurements were taken from distinct samples or whether the same sample was measured repeatedly
☐	☒	The statistical test(s) used AND whether they are one- or two-sided<br>*Only common tests should be described solely by name; describe more complex techniques in the Methods section.*
☒	☐	A description of all covariates tested
☒	☐	A description of any assumptions or corrections, such as tests of normality and adjustment for multiple comparisons
☐	☒	A full description of the statistical parameters including central tendency (e.g. means) or other basic estimates (e.g. regression coefficient) AND variation (e.g. standard deviation) or associated estimates of uncertainty (e.g. confidence intervals)
☐	☒	For null hypothesis testing, the test statistic (e.g. *F*, *t*, *r*) with confidence intervals, effect sizes, degrees of freedom and *P* value noted<br>*Give P values as exact values whenever suitable.*
☒	☐	For Bayesian analysis, information on the choice of priors and Markov chain Monte Carlo settings
☒	☐	For hierarchical and complex designs, identification of the appropriate level for tests and full reporting of outcomes
☒	☐	Estimates of effect sizes (e.g. Cohen's *d*, Pearson's *r*), indicating how they were calculated

Our web collection on statistics for biologists contains articles on many of the points above.

Software and code

Policy information about availability of computer code

Data collection	Gel image was recorded with ChemoStar Touch v0.5.65 (Intas Science Imaging). Intact mass spectrometry data were collected with Bruker maXis II ETD UHR-QToF MS - Bruker Elute UHPLC. The nano DSF melting curves were collected and analysed with PR.ThermControl v2.1.2.
Data analysis	Crystallography data were analyzed with XDS v2010 and v2021, SHELX v2001, SHARP v2.0, PHENIX 1.14 and 1.19, , COOT 0.8.6 and 0.9.8, PHASER 2.8 and 3.0, REFMAC5 5.7, ARP/wARP v7, CCP4 7.1.013, AIMLESS 0.7.4. Structure analysis was done with PyMol v2.3.2 (Schrödinger). Paired t test was done with GraphPad T test calculator (https://www.graphpad.com/quickcalcs/ttest1.cfm). Kinetics data fits were made with Origin Pro v9.1 (OriginLab). Intact mass spectrometry deconvolution was performed with Bruker Compass Data Analysis v5.1. Depictions were designed using Affinity Designer (Version 1.10.4.1198).

For manuscripts utilizing custom algorithms or software that are central to the research but not yet described in published literature, software must be made available to editors and reviewers. We strongly encourage code deposition in a community repository (e.g. GitHub). See the Nature Portfolio guidelines for submitting code & software for further information.

Data

Policy information about availability of data

All manuscripts must include a data availability statement. This statement should provide the following information, where applicable:

- Accession codes, unique identifiers, or web links for publicly available datasets
- A description of any restrictions on data availability
- For clinical datasets or third party data, please ensure that the statement adheres to our policy

DATA AVAILABILITY
All data needed to evaluate the conclusions in the article are present in the main text and/or the Supplementary Information. Structure factors and model coordinates for Lem3 have been deposited in the Protein Data Bank (PDB) under the accession code 8ANP (https://www.wwpdb.org/pdb?id=pdb_00008anp) and 8AGG (https://www.wwpdb.org/pdb?id=pdb_00008agg), for the Lem3:Rab1b complex under the accession code 8ALK (https://www.wwpdb.org/pdb?id=pdb_00008alk). Additional data related to this paper may be requested from the authors. Source data are provided with this paper.
Crystal structure data used in this study and published elsewhere are also available in the PDB, deposited with the following accession codes: 4RA2 (https://www.wwpdb.org/pdb?id=pdb_00004ra2), 6RRE (https://www.wwpdb.org/pdb?id=pdb_00006ree), 6B67 (https://www.wwpdb.org/pdb?id=pdb_00006b67), and 3NKV (https://www.wwpdb.org/pdb?id=pdb_00003nkv).

Mass spectrometry data that are not included in the source data file are avalaible upon request. These data comprise the results of enzymatic characterization for which no public repository is available. Proteomics measurements were not performed. Results derived from the mass spectrometry data are all available in the source data file.

Human research participants

Policy information about studies involving human research participants and Sex and Gender in Research.

Reporting on sex and gender	Not relevant for this study.
Population characteristics	Not relevant for this study.
Recruitment	Not relevant for this study.
Ethics oversight	Not relevant for this study.

Note that full information on the approval of the study protocol must also be provided in the manuscript.

Field-specific reporting

Please select the one below that is the best fit for your research. If you are not sure, read the appropriate sections before making your selection.

☒ Life sciences ☐ Behavioural & social sciences ☐ Ecological, evolutionary & environmental sciences

For a reference copy of the document with all sections, see nature.com/documents/nr-reporting-summary-flat.pdf

Life sciences study design

All studies must disclose on these points even when the disclosure is negative.

Sample size	Statistical predetermination of sample size was not performed. For all in vitro assays, three independent biological replicates were performed to ensure reproducibility of the findings (n=3), except for nanoDSF measurements which were performed in technical triplicates and were not used for statistical evaluation. Sample sizes were chosen by following the rule of providing three independant biological or technical replicates, depending on the experiment as mentioned in the manuscript for all individual experiments. This sample size was considered to be sufficient since no outliers were detected.
Data exclusions	No data were excluded.
Replication	All data presented in the manuscript are reproducible. Numbers of replications for each experiment are stated in the corresponding figure.
Randomization	Randomization was not possible for the experiments described herein and was thus not relevant for this study.
Blinding	Blinding was not possible for the experiments described herein and was thus not relevant for this study.

Reporting for specific materials, systems and methods

We require information from authors about some types of materials, experimental systems and methods used in many studies. Here, indicate whether each material, system or method listed is relevant to your study. If you are not sure if a list item applies to your research, read the appropriate section before selecting a response.

Materials & experimental systems

n/a	Involved in the study
☒	☐ Antibodies
☒	☐ Eukaryotic cell lines
☒	☐ Palaeontology and archaeology
☐	☒ Animals and other organisms
☒	☐ Clinical data
☒	☐ Dual use research of concern

Methods

n/a	Involved in the study
☒	☐ ChIP-seq
☒	☐ Flow cytometry
☒	☐ MRI-based neuroimaging

Animals and other research organisms

Policy information about studies involving animals; ARRIVE guidelines recommended for reporting animal research, and Sex and Gender in Research

Laboratory animals	Escherichia coli (strains: Mach1, BL21-CodonPlus (DE3)-RIL, BL21-CodonPlus (DE3))
Wild animals	Not relevant for this study.
Reporting on sex	Not relevant for this study.
Field-collected samples	Not relevant for this study.
Ethics oversight	Not relevant for this study.

Note that full information on the approval of the study protocol must also be provided in the manuscript.
